# Cu^2+^/Cu^+^ redox-cycling-driven dual-mode sensor for simultaneous monitoring of acetylcholinesterase activity and pesticide exposure

**DOI:** 10.3389/fphar.2025.1640821

**Published:** 2025-08-22

**Authors:** Sitong Lai, Kunhui Sun, Yun Wu, Xueyuan Wu, Yiqi Yan, Guojing Liu, Xiaoyi Liu, Yuanyuan Ge, Lina Zeng, Ziyu Guo, Shuhong Wang, Ping Wang, Bing Wang, Han Zhang, Xie-an Yu

**Affiliations:** ^1^ Institute of Traditional Chinese Medicine, Tianjin University of Traditional Chinese Medicine, Tianjin, China; ^2^ Shenzhen Institute for drug Control, Shenzhen, China; ^3^ Shenzhen tsumura medicine Co., Ltd., Shenzhen, China; ^4^ Jiangmen Institute for Drug Control, Jiangmen, China; ^5^ Haihe Laboratory of Modern Chinese Medicine, Tianjin, China

**Keywords:** traditional Chinese medicines, organophosphorus pesticides, acetylcholinesterase, redox-cycling-driven, dual-mode sensor

## Abstract

**Introduction:**

The procedural complexity and time-consuming of conventional pesticide residue detection methods in traditional Chinese medicines (TCMs) significantly impeded their application in modern systems. To address this, this study presented an innovative dual-mode sensor driven by Cu^2+^/Cu^+^ redox-cycling, which achieved efficient signal transduction from enzyme inhibition to optical response for rapid acetylcholinesterase (AChE) activity and organophosphorus pesticide (OP) residue detection.

**Methods:**

The AB-Cu NPs sensor, a dynamic redox-responsive system, was constructed via coordination-driven assembly of Azo-Bodipy 685 (AB 685) and Cu^2+^. Initially, Cu^2+^ quenched the optical signals of AB 685 through photoinduced electron transfer (PET), maintaining an “OFF” state. Upon AChE-catalyzed hydrolysis of acetylthiocholine chloride (ATChCl) to thiocholine (TCh), Cu^2+^ was reduced to Cu^+^, thereby activating dual ultraviolet-visible (UV-Vis)/fluorescence (FL) signals (“ON” state). OP residues were quantified by their inhibition of AChE, which blocked Cu^2+^/Cu^+^ conversion and suppressed signal generation.

**Results:**

The sensor exhibited positive sensitivity with detection limits of 0.0327 U/L for AChE activity and 1.72 ng/mL for triazophos, leveraging Cu^2+^/Cu^+^ redox-cycling for signal amplification. Notably, the Cu^2+^/Cu^+^ valence interconversion coupled enzyme inhibition with probe responsiveness, ensuring the both procedural easily and rapid response.

**Discussion:**

This study developed a portable detection platform based on valence interconversion coupled enzyme inhibition with probe responsiveness. The dual-signal output enhanced reliability and the redox-driven mechanism enabled signal amplification. This advancement provided a real-time, portable and rapid detection for AChE activity and pesticide exposure of TCMs, bridging gaps in agricultural safety and pharmaceutical standardization.

## 1 Introduction

The global recognition of traditional Chinese medicine (TCM) has driven an exponential growth in the demand for medicinal herbs, resulting in severe depletion of wild resources (Xiao et al., 2019; Tripathy et al., 2015). To meet industrial-scale cultivation needs, chemical pesticides are extensively employed to regulate plant growth and combat pathogens (Xiao et al., 2019). However, pesticide residues in herbal matrices not only compromise medicinal quality but also introduce “secondary pharmaceutical contamination” with latent health risks (Tripathy et al., 2015; Zhang et al., 2012). Of particular concern are organophosphorus pesticides (OPs), whose chronic low-dose exposure may induce carcinogenicity, endocrine disruption, and immunosuppression, thereby contradicting the fundamental principle of “safety-efficacy” in TCM systems (Jung et al., 2022; Ubaid Ur Rahman et al., 2021; Falfushynska et al., 2022). Consequently, developing analytical platforms for OP detection is imperative to ensure the sustainable globalization of TCM.

Although current mainstream chromatographic techniques (gas chromatography, GC; high-performance liquid chromatography, HPLC) demonstrate high detection accuracy, their laborious sample pretreatment procedures and protracted analysis time severely constrain their practical applicability in field settings (Bhattu et al., 2021; Correa et al., 2020; Acosta-Dacal et al., 2021; Peng et al., 2017). These limitations have spurred innovations in biosensing strategies, particularly acetylcholinesterase (AChE)-inhibition-based approaches and methods of fluorescence (FL) probes (Cai et al., 2021; Jiang et al., 2025; Wang et al., 2024; Alex and Mukherjee, 2021). OPs irreversibly inhibit AChE activity, blocking the enzymatic hydrolysis of acetylthiocholine chloride (ATChCl) into thiocholine (TCh) and acetic acido, thereby enabling indirect OP quantification via TCh production (Jiang et al., 2025; Alex and Mukherjee, 2021; Korram et al., 2019). Nevertheless, current enzyme inhibition methods cannot enable simultaneous monitoring directly. Conversely, FL probes enable simultaneous monitoring but suffer from inherent limitations including inadequate sensitivity and elevated detection thresholds (Tang et al., 2024; He et al., 2022; Ullah et al., 2025). To transcend the limitations of conventional single-mode detection mechanisms, the synergistic integration of enzymatic inhibition with FL probe into a multi-mode sensing platform held significant promise for concurrent achieving of both procedural easily and rapid response. Shixian Zhao et al. designed a dual-mode detection system based on the enzyme inhibition method using FL materials, achieving high sensitivity (Zhao et al., 2025). Yin Dai et al. synthesized BSA-CeO_2_ NCs, which combined the enzyme-like catalytic properties of BSA-CeO_2_ NCs with their fluorescence properties to achieve multimodal sensing of analytes (Dai et al., 2024). De Yan Li et al. developed a metal ions-mediated signal amplification strategy for the assaying AChE activity and screening its inhibitors (Li et al., 2024). However, these sensors still suffer from prolonged fabrication time or reliance on the traditional Ellman’s assay principle, which entails complex operations and consequently time-consuming procedures. Therefore, time-efficient sensors are highly desirable.

**SCHEME 1:**
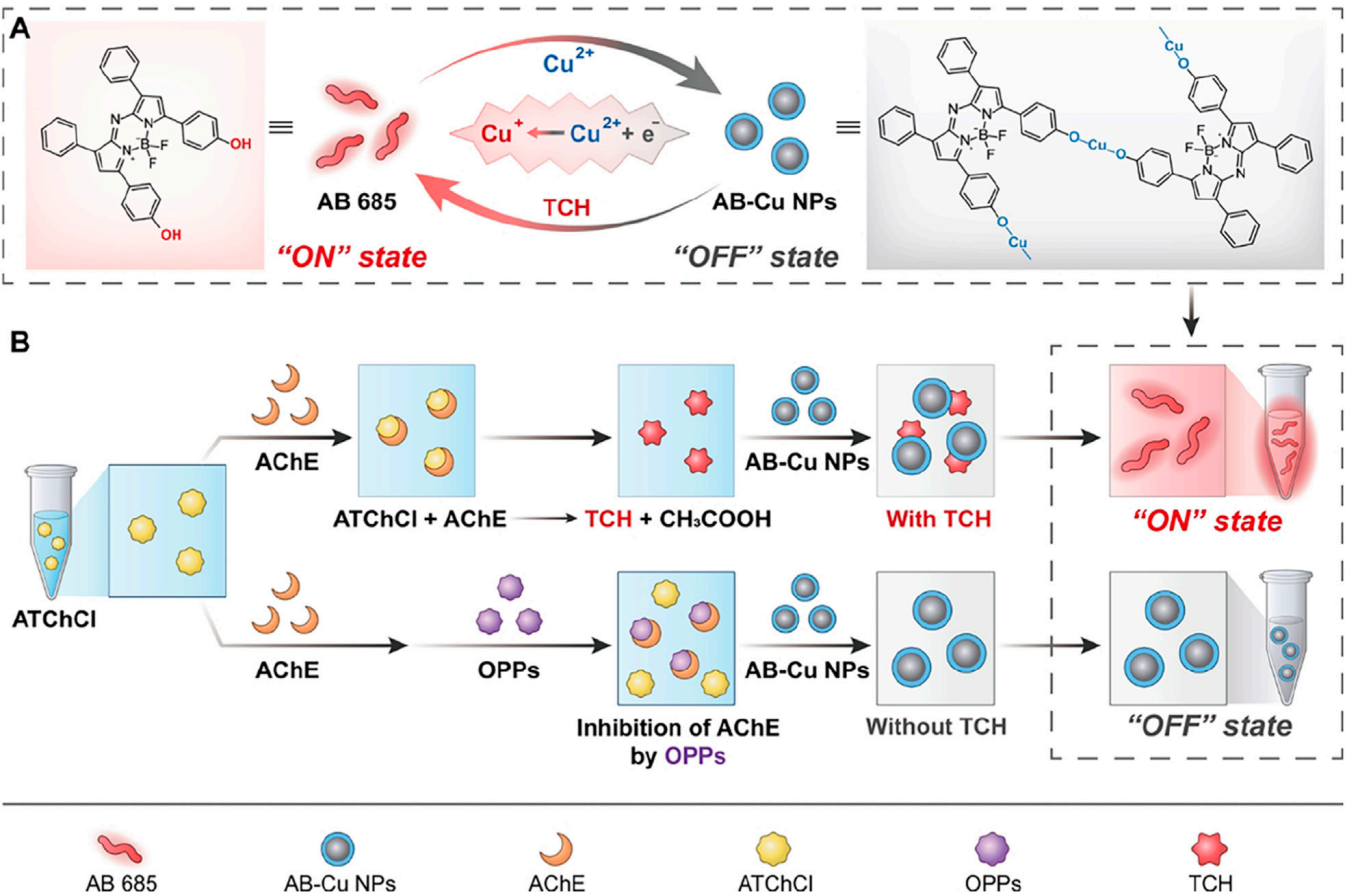
**(A)** Schematic of the Cu^2+^/Cu^+^ redox-cycling-driven sensor. Cu^2+^ quenches AB 685 optical signals, maintaining the sensor in the “OFF” state. Upon addition of TCh, Cu^2+^ is reduced to Cu^+^, resulting in optical signals recovery and the “ON” state. **(B)** Dual-mode detection of AChE activity and OPPs: AChE catalyzes the hydrolysis of ATChCl to TCh, which activates the sensor to an “ON” state. When AChE is inhibited by OPPs, TCh is not produced, switching the sensor to an “OFF” state.

In this study, we developed a novel sensor (AB-Cu NPs) formed by coordination-driven assembly with Azo-Bodipy 685 (AB 685) and Cu^2+^ via integrating enzymatic inhibition with FL/Ultraviolet-visible (UV-Vis) dual-mode detection, thereby establishing an efficient analytical platform for OP residue screening in TCMs (Scheme 1). Specifically, Cu^2+^ acted as highly efficient electron acceptor, effectively quenching the FL of the AB 685 through photoinduced electron transfer (PET), which led to the formation of homogeneous and stable complex nanoparticles (AB-Cu NPs). At this phase, Cu^2+^ quenched the optical signals to maintain an “OFF” state. During the AChE-catalyzed hydrolysis of ATChCl to TCh, Cu^2+^ was reduced to Cu^+^, thereby activating dual UV-Vis/FL signals (“ON” state). By inhibiting the activity of AChE, OPs blocked Cu^2+^/Cu^+^ conversion, thereby enabling residue quantification via signal suppression-mediated “OFF” state recovery.

The proposed sensor exhibited three groundbreaking features: (1) Near-infrared optical properties: The AB 685 exhibited distinct advantages in the near-infrared (NIR) spectral region, featuring a UV-Vis absorption peak at 685 nm and FL emission spanning 710–730 nm, which effectively mitigated matrix interference, while dual-mode cross-validation enhanced signal reliability; (2) Dihydroxy-enabled “ON-OFF-ON” switching mechanism: AB 685 undergone oxidative coordination with Cu^2+^ to form AB-Cu NPs, resulting in characteristic absorption attenuation and FL quenching. The subsequent introduction of AChE-catalyzed reductive TCh triggered AB-Cu NPs disassembly and optical signal recovery; (3) Quantitative OP detection: OPs inhibited AChE activity, reducing TCh production and thereby diminishing sensor signal restoration in a concentration-dependent manner. This redox-mediated optical switching strategy pioneers a rapid-response solution for quality assurance in TCM supply chains.

## 2 Experimental section

### 2.1 Instruments

UV-Vis absorption and FL spectra were acquired using a Varioskan Flash multimode microplate reader (3001, Thermo Fisher Scientific, Vantaa, Finland). Enzyme incubation was performed in a biochemical incubator (KB53, BINDER GmbH, Tuttlingen, Germany). Reaction processes were conducted using a constant-temperature mixer (BE-3500, Qilinbeier Instrument Manufacturing Co., Ltd., Haimen, China). Vortex mixing was performed using a benchtop mixer (VORTEX-3, Xirun Scientific Instruments Co., Ltd., Shanghai, China). Morphological characterization was carried out using a Talos F200X G2 transmission electron microscope (Thermo Fisher Scientific, Hillsboro, OR, USA). FL imaging was conducted using an *in vivo* imaging system (Model IVIS Lumina III, PerkinElmer, Waltham, MA, USA). Nanoparticle size distribution and zeta potential measurement were performed using a Zetasizer Nano ZS90 dynamic light scattering analyzer (Malvern Panalytical Ltd., Malvern, UK). Ultrapure water (18.2 MΩ cm) was prepared using a Millipore Simplicity water purification system (Merck Millipore, MA, USA).

### 2.2 Chemical reagents and materials

Azo-Bodipy 685 (AB 685) was obtained from Xi’an Ruixi Biological Technology Co., Ltd. (Xi’an, China). Cupric chloride dihydrate (CuCl_2_·2H_2_O, ≥99.99% purity) was purchased from Shanghai Aladdin Biochemical Technology Co., Ltd. (Shanghai, China). HPLC-grade acetonitrile (CH_3_CN, ≥99.9%) was acquired from Macklin Biochemical Co., Ltd. (Shanghai, China). Acetylcholinesterase (AChE, from fly head, 210 U/g) was procured from MedChemExpress LLC (Monmouth Junction, NJ, USA). Acetylthiocholine chloride (ATChCl, ≥99.0% purity) was obtained from Shanghai Aladdin Biochemical Technology Co., Ltd. Certified reference materials including triazophos standard solution (100 μg/mL in methanol), chlorpyrifos standard solution (100 μg/mL in acetonitrile), oxychlordane standard solution (100 μg/mL in hexane), *cis*-chlordane standard solution (100 μg/mL in hexane), heptachlor exo-epoxide standard solution (100 μg/mL in hexane) were supplied by Alta Scientific Co., Ltd. (Tianjin, China). Sterile physiological saline (0.9% w/v NaCl solution) was purchased from Beyotime Biotechnology (Shanghai, China). L-Ascorbic acid (vitamin C, ≥98.0% purity) was acquired from Shanghai Aladdin Biochemical Technology Co., Ltd. All chemicals were of analytical reagent grade or higher purity unless otherwise stated.

### 2.3 UV-Vis and FL spectra of AB 685

A 1 μL aliquot of 10 mM AB 685 solution in acetonitrile (CH_3_CN) was dissolved in 80 μL of solvents with varying organic-aqueous ratios (0% (pure water), 10% (v/v), 30% (v/v), 50% (v/v), 70% (v/v), 90% (v/v), and 100% (pure CH_3_CN) CH_3_CN in water). UV-Vis absorption and FL spectra of the AB 685 were recorded in these solvent systems.

### 2.4 Synthesis of AB-Cu NPs

A 80 μL 50% CH_3_CN solution mixture of 39.5 μL CH_3_CN, 38.5 μLwater, 0.5 μL of 10 mM AB 685 solution and 1.5 μL of 50 mM CuCl_2_ aqueous solution were added. The mixture was vortexed vigorously for 5 s at room temperature under airtight conditions to obtain AB-Cu NPs.

### 2.5 Characterization of AB-Cu NPs

The AB-Cu NPs was characterized by morphology analysis, hydrodynamic size, zeta potential, UV-Vis absorption and FL spectra. AB-Cu NPs were dropped onto a carbon-coated copper grid for TEM measurement. DLS were performed on a Malvern Zetasizer Nano-Z instrument. And UV-Vis absorption and FL spectra were recorded using a microplate reader. All determinations were performed at least three times.

### 2.6 Validation of “ON-OFF-ON” of AB-Cu NPs

Vitamin C was used to validate the dual-mode response of AB-Cu NPs. UV-Vis absorption spectra as well as color change of the sample solution, FL spectra and FL imaging were conducted to verify “ON-OFF-ON” of AB-Cu NPs, with the AB 685 as the negative control, AB-Cu NPs co-incubated with vitamin C as the positive.

### 2.7 Detection of AChE activity using AB-Cu NPs

10 μL of AChE solution was incubated with 20 μL of 0.9% saline at 37 °C for 10 min to activate the enzyme. Subsequently, 20 μL of 50 mM ATChCl was added, followed by incubation for 30 min. The reaction was terminated by adding 50 μL of CH_3_CN. Next, 40 μL of the reaction mixture was mixed with 40 μL of the AB-Cu NPs and vortexed for 20 min at room temperature. UV-Vis absorption (685 nm) and FL emission (720 nm) intensities were measured to quantify AChE activity. Among them, the final reaction system volume was 80 μL, with AChE concentrations ranging from 0 U/L to 600 U/L. All solutions were stored at −4 °C and used within 7 days.

### 2.8 Detection of OPs using AB-Cu NPs

A mixture of 1 μL triazophos solution and 10 μL AChE solution was added to a microcentrifuge tube containing 19 μL of 0.9% saline. The mixture was incubated at 37 °C for 20 min, followed by the addition of 20 μL ATChCl (50 mM) and further incubation for 30 min. The reaction was terminated by adding 50 μL acetonitrile. And the other procedures were the same as described above (Section 2.7). Finally, the UV-Vis absorption and FL emission signal intensities were measured to determine the triazophos content. Among them, the final reaction volume was adjusted to 80 μL with triazophos concentrations ranging from 0.05 μg/mL to 5 μg/mL (UV mode) and 0.01 μg/mL to 5 μg/mL (FL mode).

Likewise, the operation procedures of the chlorpyrifos were same to that of triazophos. And the chlorpyrifos content was determined via measuring the UV-Vis absorption and FL emission signal intensities. Among them, the final reaction volume was adjusted to 80 μL with chlorpyrifos concentrations ranging from 0.01 μg/mL to 5 μg/mL (UV/FL mode). FL intensity at 720 nm and UV-Vis absorption at 685 nm were recorded. Oxychlordane, *cis*-chlordane, heptachlor exo-epoxide were selected as negative controls. All solutions were stored at −4 °C until further use.

### 2.9 Real sample analysis

To evaluate the sensor’s anti-interference capability in complex matrices, *Citri Reticulatae Pericarpium* (Chenpi) extract was prepared according to the *Chinese Pharmacopoeia*. Briefly, 0.2 g of sample was extracted with 25 mL methanol. Triazophos stock solution was spiked into the extract to achieve final concentrations of 0.05 μg/mL, 0.1 μg/mL and 0.5 μg/mL. The sensor was used to determine pesticide residues following the procedure in Section 2.8 and recovery rates were calculated. Additionally, ten batches of Chenpi samples (labeled as No. 6, 8, 9, 10, 94, 95, 96, 97, 99, 100) were randomly sprayed with varying concentrations of triazophos pesticide in a double-blind experiment.

At the same time, fifteen batches of Chenpi samples named S2410050, S2409650, S2407290, S2406550, S2406090, S2405170, S2404720, S2404830, S2404160, S2403480, S2403340, S2403100, S2402270, S2402010 and S2402020 were analyzed for triazophos pesticide residues. Sample extracts were prepared according to the *Chinese Pharmacopoeia* standard protocols. The obtained extracts were concentrated at controlled low temperatures. Subsequent analysis was performed by AB-Cu NPs under FL mode. All sample solutions were aliquoted into sealed vials and preserved in refrigeration unit maintained at 4 °C under for storage.

## 3 Results and discussion

### 3.1 Characterization of AB 685

**FIGURE 1:**
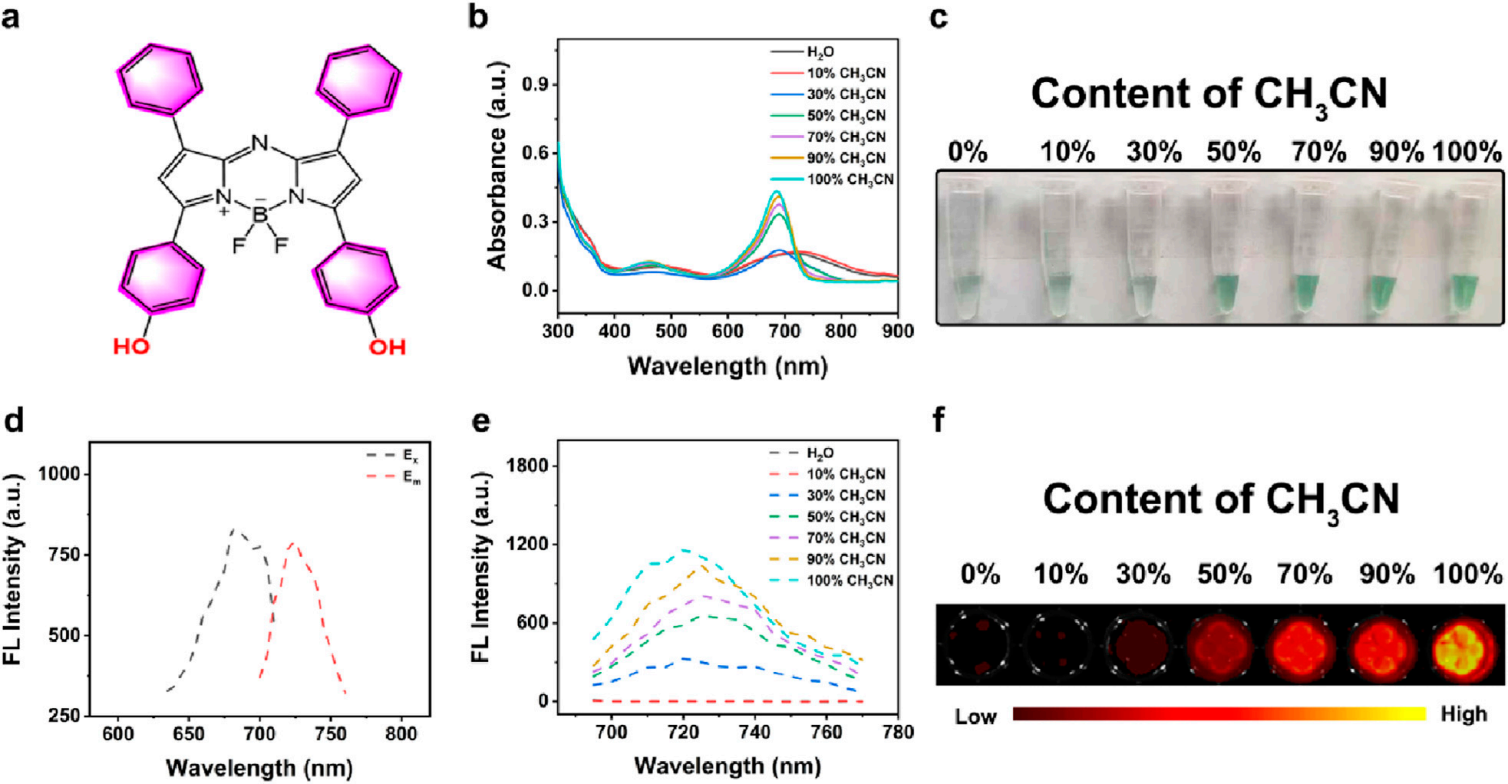
**(a)** The structural formula of AB 685. **(b)** UV-Vis spectra of AB 685 in aqueous and CH_3_CN mixed solutions with varying volumetric ratios: 0% (pure H_2_O), 10% (v/v), 30% (v/v), 50% (v/v), 70% (v/v), 90% (v/v), and 100% (pure CH_3_CN). **(c)** Bright-field images of AB 685 in different solvent systems. **(d)** Excitation and emission spectra of AB 685. **(e)** FL emission profiles of AB 685 across solvent gradients. **(f)** FL imaging of AB 685 in mixed solvents.

BODIPY (boron-dipyrromethene) probes represent a class of fluorescent dyes with unique photophysical properties, which have gained considerable attention in bioimaging, chemical sensing due to their exceptional photochemical stability and high quantum yield (Loudet and Burgess, 2007; Ulrich et al., 2008; Boens et al., 2012; Yan et al., 2022). As shown in Figure 1a, AB 685 was a BODIPY-based FL probe containing bisphenol hydroxyl groups. To optimize the AB 685s application for enzyme activity and pesticide residue detection, we systematically examined the UV-Vis absorption and FL emission spectra of AB 685 in various organic-aqueous solvent systems. AB 685 exhibited a distinct absorption peak between 600–800 nm and a pronounced blue shift in the absorption maxima was observed with increasing organic solvent content, accompanied by a gradual enhancement in absorption intensity at 685 nm. Notably, the absorption peak at 685 nm within the aqueous solution containing 50% acetonitrile showed a significant intensification compared to that within the aqueous solution containing 30% acetonitrile (Figure 1b). Concurrently, AB 685 demonstrated visually observable color changes corresponding to the UV-Vis absorption intensity variations - the solution exhibited progressively intensified green coloration with increasing organic solvent content (Figure 1c).

AB 685 possessed excellent fluorescent properties with excitation and emission maxima at 685 nm and 720 nm, respectively (Figure 1d). The FL intensity of the AB 685 also exhibited a positive correlation with organic solvent content, showing particularly significant enhancement at 50% organic solvent compared to 30% (Figure 1e). FL imaging further confirmed the above trend, demonstrating negligible FL in purely aqueous solution but progressively intensified emission with increasing organic solvent proportion (Figure 1f). Based on these results, we selected the aqueous solution containing 50% acetonitrile for subsequent experiments to maximize the dual-mode (UV-Vis/FL) detection capability of AB 685 for pesticide residue analysis.

### 3.2 Characterization of AB-Cu NPs

**FIGURE 2:**
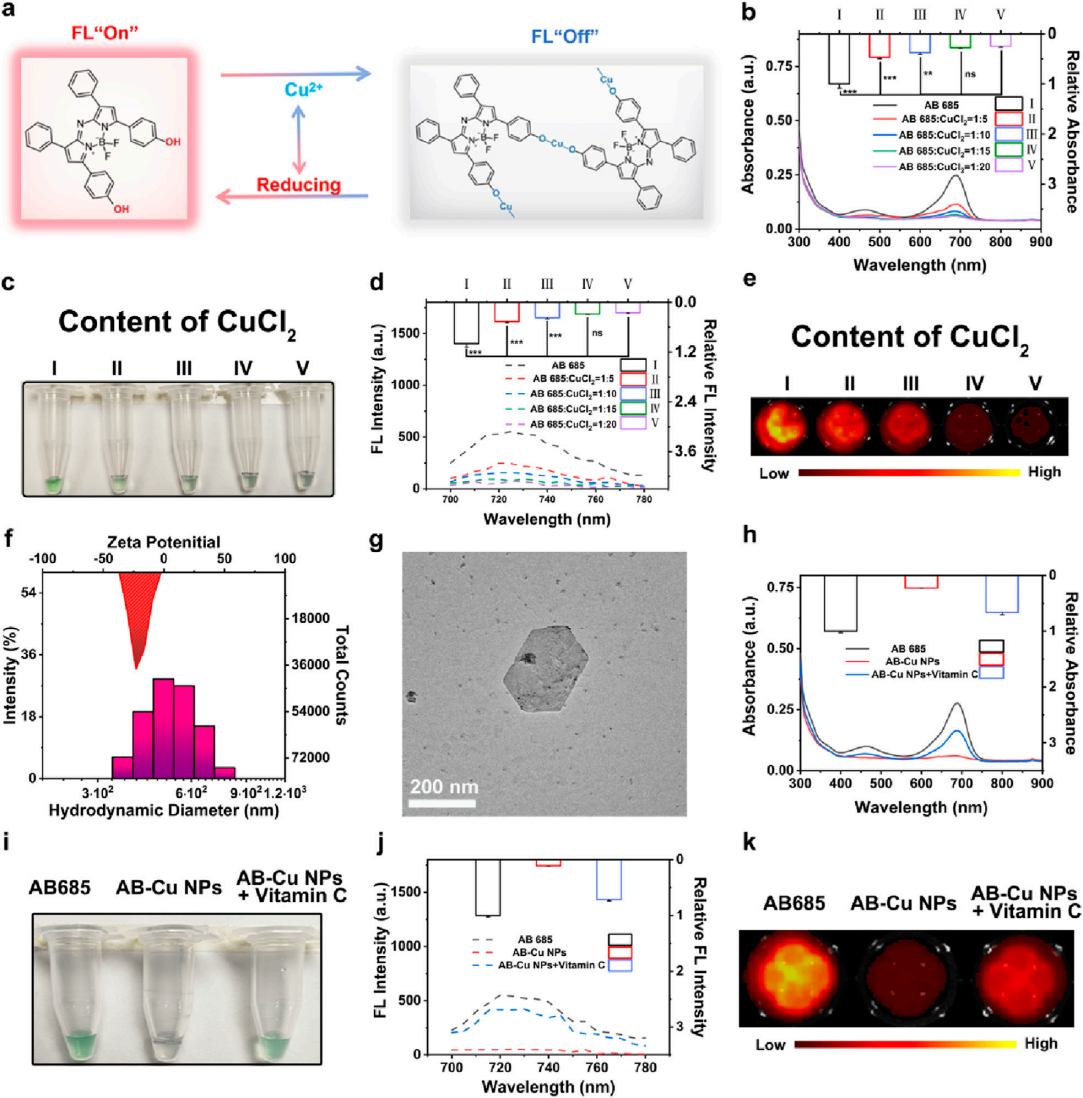
**(a)** Schematic illustration of the response mechanism of AB-Cu NPs. **(b)** UV-Vis absorption spectra of AB-Cu NPs prepared with varying molar ratios of AB 685 to Cu^2+^ (1:0, 1:5, 1:10, 1:15, 1:20). Data are presented as mean ± SD (n = 3; **p < 0.01, ***p < 0.001). **(c)** Bright-field images corresponding to the systems in **(b)**. **(d)** FL emission spectra of AB-Cu NPs with increasing Cu^2+^ concentrations (molar ratios: 1:0 to 1:20). Data are presented as mean ± SD (n = 3; ***p < 0.001). **(e)** FL images corresponding to the systems in **(d)**. **(f)** DLS analysis of AB-Cu NPs. **(g)** TEM image of AB-Cu NPs (Scale bar: 200 nm). **(h)** Comparative UV-Vis spectra of AB 685, AB-Cu NPs, AB-Cu NPs + Vitamin **(c) (i)** Bright-field visualization corresponding to the systems in **(h)**. **(j)** FL spectra of AB 685, AB-Cu NPs, and AB-Cu NPs + Vitamin **(c) (k)** FL images corresponding to the systems in **(j)**. Abbreviation: ns, not significant.

As evidenced by mechanistic studies, that Cu^2+^ as an efficient electron acceptor, could effectively capture excited-state electrons through electron energy transfer mechanisms, forming stable complexes accompanied by the change of optical phenomenon (Wang et al., 2022). Based on the distinctive dihydroxy structure of AB 685 and the electron-capturing property of Cu^2+^, we developed the AB-Cu NPs sensor via oxidative coordination between Cu^2+^ and AB 685. As schematically illustrated in Figure 2a, the mechanism of sensor relied on the reductive conversion of Cu^2+^ within the AB-Cu NPs framework, leading to structural disintegration of the AB-Cu NPs and subsequent release of AB 685, which triggered the recovery of both UV-Vis absorption and FL emission signals.

The quenching of optical signals AB-Cu NPs involved three key mechanisms: (1) Cu^2+^ acted as an efficient electron acceptor through PET; (2) Cu^2+^ coordinated with AB 685 to form stable complexes, directly altering the electronic structure of the AB 685; (3) Nanoparticle formation induced aggregation of AB 685 molecules, leading to aggregation-caused quenching (ACQ) effects (Korram et al., 2019; Wang et al., 2022; Wang et al., 2023; Chen et al., 2020; Herten et al., 2018; Hu et al., 2018). Spectroscopic analyses demonstrated that increasing Cu^2+^ concentration caused progressive reduction in both absorption intensity and FL emission of AB 685, accompanied by fading of the green coloration and formation of black complexes (Figures 2b–e). Among them, optimal sensor preparation was achieved at n_(AB 685)_:n(_Cu_^2+^) = 1:15, as determined by both UV-Vis absorption and FL intensity changes. AB-Cu NPs were synthesized within only 5 s through a green process requiring minimal materials.

DLS measurements confirmed fine dispersion of AB-Cu NPs with a hydrodynamic diameter of 488.7 nm, while zeta potential of −23.11 mV indicated excellent colloidal stability of AB-Cu NPs (Figure 2f). TEM imaging revealed that AB-Cu NPs had a well-dispersed polygonal nanostructures (Figure 2g; Supplementary Figure S1), illustrating the successful synthesis of AB-Cu NPs. The AB-Cu NPs exhibited excellent stability, with negligible variations in hydrodynamic diameter and FL intensity observed over a 7-day period (Supplementary Figures S2-S3).

To evaluate the dual-mode detection capability of AB-Cu NPs based on redox-cycling, vitamin C was used as a reducing agent for validation. Upon reduction, the system exhibited significant restoration of AB 685s characteristic UV-Vis absorption intensity and the green coloration (Figures 2h,i). Similarly, FL spectra and imaging confirmed recovery of the quenched FL signal (Figures 2j,k). These above results demonstrated the successful fabrication of a UV-Vis/FL dual-mode “ON-OFF-ON” sensor through simple coordination between the AB 685 and Cu^2+^.

### 3.3 Validation of AB-Cu NPs’ response to AChE

**FIGURE 3:**
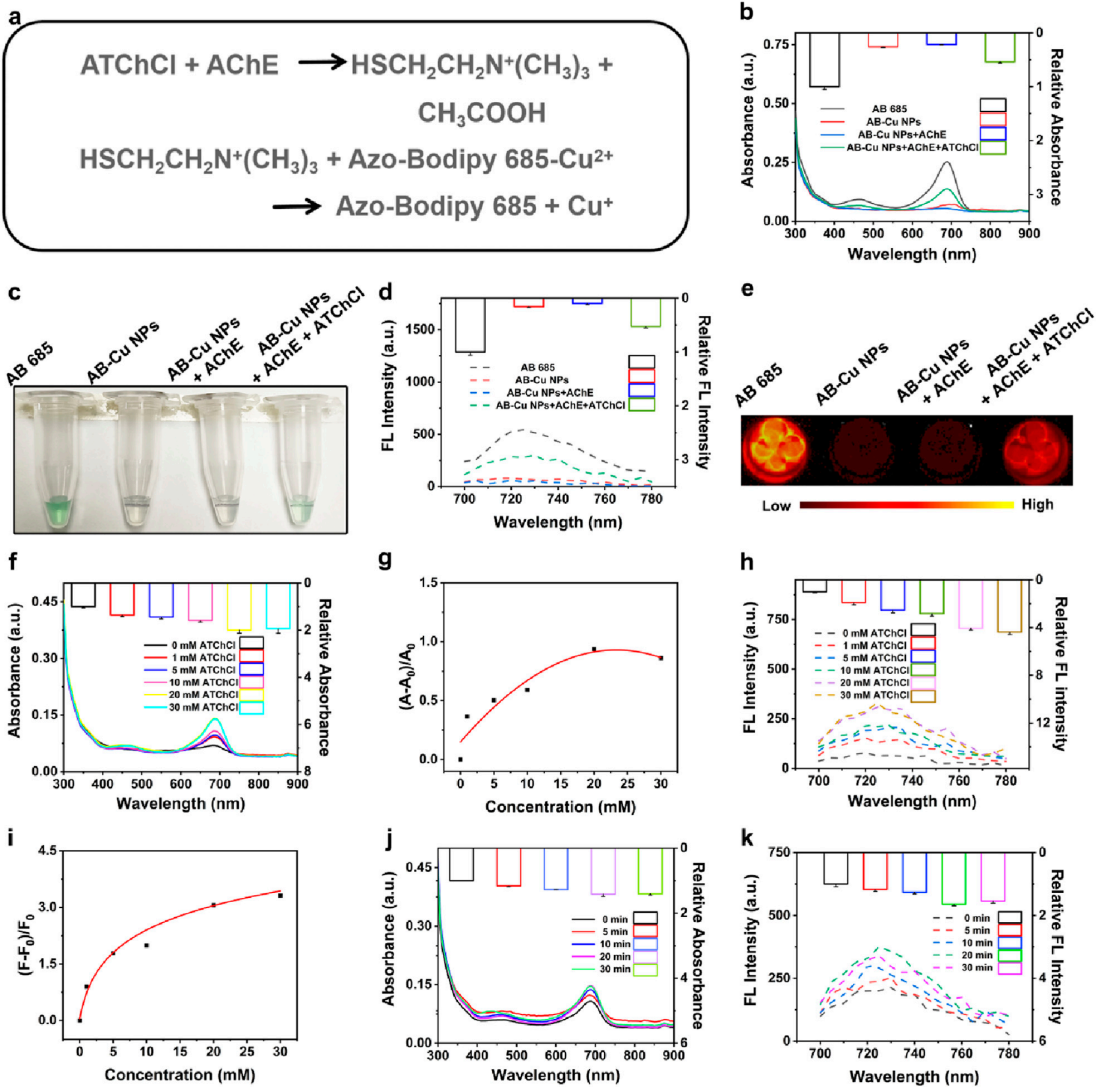
**(a)** Reaction equations of AChE with ATChCl and subsequent interactions with AB-Cu NPs. **(b)** Comparative UV-Vis absorption spectra of AB 685, AB-Cu NPs, AB-Cu NPs + AChE, and AB-Cu NPs + AChE + ATChCl systems. **(c)** Bright-field microscopic visualization corresponding to the systems in **(b)**. **(d)** FL emission spectra of AB 685, AB-Cu NPs, and their enzyme-modulated states. **(e)** FL images of the corresponding systems shown in **(c)**. **(f)** UV-Vis absorption spectra under varying ATChCl concentrations. **(g)** Relative UV-Vis absorption changes versus ATChCl concentration. **(h)** FL emission spectra at different ATChCl concentrations. **(i)** Concentration-dependent FL intensity variations. **(j)** Time-dependent UV-Vis absorption spectra. **(k)** Time-dependent FL emission spectra.

As established in previous sections, the AB-Cu NPs exhibited responsiveness to reducing vitamin C. Notably, the enzymatic reaction between AChE and its substrate ATChCl yielded TCh, a reductant containing a sulfhydryl (-SH) group capable of reducing Cu^2+^ to Cu^+^ within the sensor matrix. This redox process triggered UV-Vis and FL recovery of the AB-Cu NPs, thereby enabling quantitative assessment of AChE activity (see Reaction Figure 3a). Systematic validation was performed through multiple analytical approaches, including UV-Vis spectrophotometry, bright-field microscopy, FL intensity and FL imaging. These results demonstrated that the TCh indeed restored UV-Vis absorption intensity, accompanied by the recovery of the solution’s green color and FL intensity, along with visible FL imaging changes (Figures 3b–e). Meanwhile, the AChE-only group served as a negative control. These findings indicated that the AB-Cu NPs could monitor the reaction progress between AChE and its substrate, achieving the detection of AChE activity. Meanwhile, sensor performance was evaluated by monitoring FL variation under TCh-mediated reduction across different storage durations. Throughout the 7-day testing window, the FL recovery efficiency maintained no obvious change (Supplementary Figure S4).

To further promote the reaction sensitivity, the AChE and ATChCl reaction system was systematically optimized with respect to two critical parameters: substrate concentration and sensor-reaction product interaction time. UV-Vis spectral analysis revealed a concentration-dependent increase in absorption intensity, reaching maximum signal amplitude at 20 mM ATChCl (Figures 3f,g; Supplementary Figures S5-S6). Further elevation of ATChCl concentration (>20 mM) resulted in slight signal attenuation, potentially attributable to molecular crowding effects that impede reactant diffusion and hinder reaction kinetics. Parallel FL measurements exhibited consistent behavior, with signal saturation occurring beyond 20 mM ATChCl (Figures 3h,i; Supplementary Figures S7-S8). Consequently, 20 mM ATChCl was established as the optimal substrate concentration for subsequent experiments.

The optical signal restoration mechanism relied on the redox reaction between AB-Cu NPs and enzymatically generated TCh. The temporal analysis revealed that the sensor exhibited an instantaneous response upon encountering the reaction product at t = 0 min. And the optical signal continued to gradually increase with incubation time, reaching its maximum intensity at 20 min (Figures 3j,k). Therefore, the optimal reaction time was determined to be 20 min. Notably, the instantaneous response observed at t = 0 min demonstrated that the AB-Cu NPs possessed rapid response capability for the detection of AChE.

### 3.4 Detection of AChE activity using AB-Cu NPs

**FIGURE 4:**
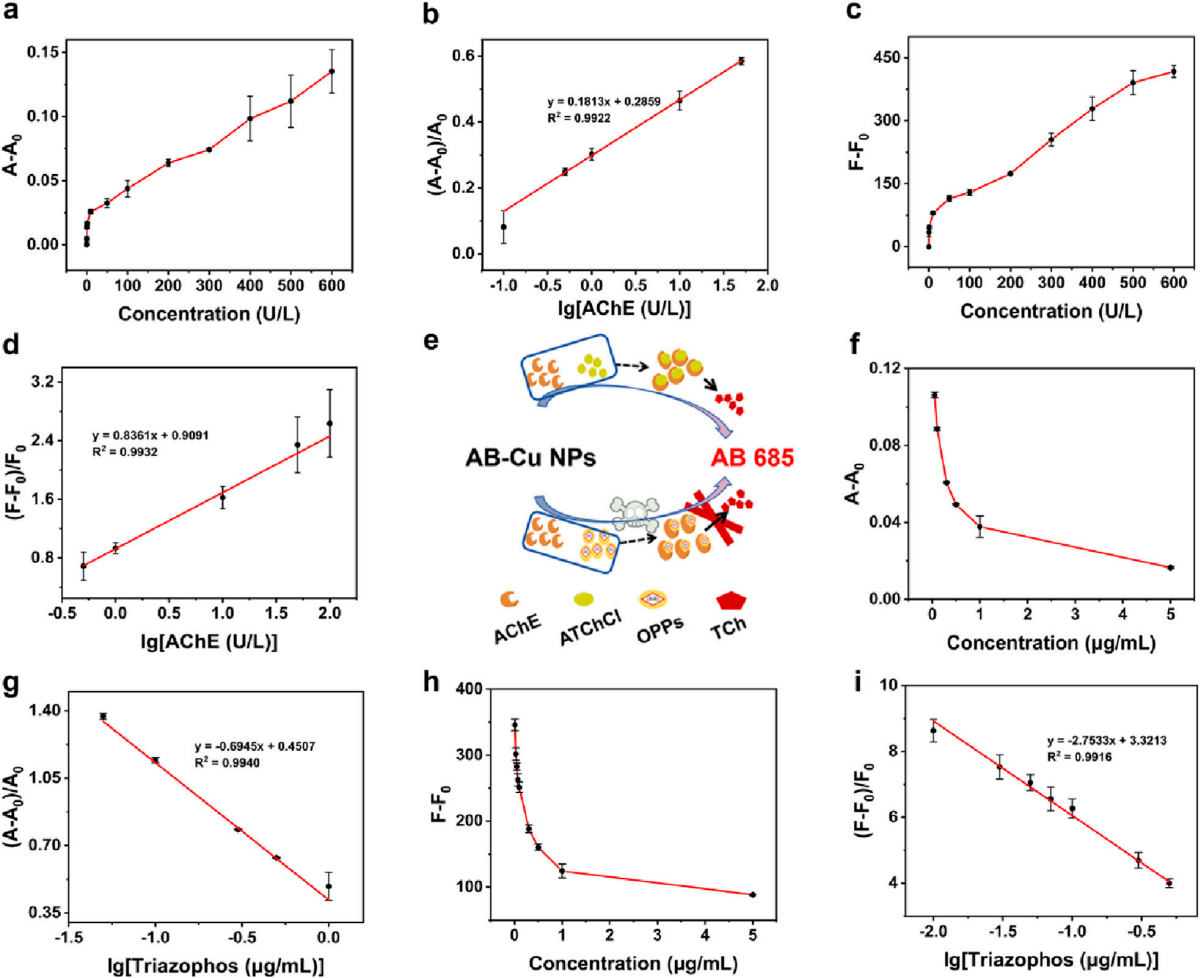
**(a)** UV-Vis absorption intensity variations at varying AChE concentrations. **(b)** Calibration curve of AChE based on UV-Vis absorption. **(c)** FL intensity changes under different AChE concentrations. **(d)** FL-based calibration curve for AChE. **(e)** Schematic of detection for AChE activity and OPPs. **(f)** UV-Vis absorption variations responses to triazophos pesticide concentrations. **(g)** UV-Vis-derived calibration curve for triazophos. **(h)** Concentration-dependent FL variations of triazophos. **(i)** FL calibration curve for triazophos pesticide.

Following optimization of the concentration of ATChCl and reaction time, the AB-Cu NPs was employed for “dual-mode” quantitative analysis of AChE activity. These results revealed systematic changes in the maximum UV-Vis absorption peak intensity and optimal FL emission intensity with variations in TCh concentration. This concentration-dependent recovery of optical signals originated from the interaction between the enzymatic reaction product of AChE and AB-Cu NPs, which triggered distinct optical responses. Within the AChE concentration range of 0–600 U/L, the UV-Vis absorption intensity progressively recovered and increased. Notably, a linear relationship was observed between the logarithm of AChE concentration (0.1–50 U/L) and the proportional change in UV-Vis absorption, described by the equation $\frac{A-A0}{A0}$ = 0.1813 × log[AChE] + 0.2859 (R^2^ = 0.9922), with a limit of detection (LOD) of 0.0327 U/L (Figures 4a,b). Similarly, the FL intensity of the sensor gradually intensified as AChE activity increased from 0 U/L to 600 U/L. A linear correlation was established between the logarithm of AChE concentration (0.5–100 U/L) and the maximum FL intensity, following the equation $\frac{F-F0}{F0}$ = 0.8361 × log[AChE] + 0.9091 (R^2^ = 0.9932), with a LOD of 0.0861 U/L (Figures 4c,d). These findings demonstrated that both detection modes of the sensor enable quantitative analysis of AChE activity.

### 3.5 Detection of OPs using AB-Cu NPs

To validate the sensor’s response sensitivity to OPs, triazophos and chlorpyrifos were utilized to inhibit AChE activity while monitoring the corresponding variations in AB-Cu NPs signals, the schematic was illustrated in Figure 4e. Oxychlordane, *cis*-chlordane, heptachlor exo-epoxide were selected as negative controls. The results demonstrated that triazophos and chlorpyrifos significantly inhibited AChE activity, consequently suppressing FL recovery. In contrast, non-organophosphorus pesticides oxy-chlordane et al. showed no interference with FL recovery (Supplementary Figure S9). These findings establish the foundation for AB-Cu NPs based detection of OP residues. The calibration curves were established between triazophos concentration and the differential parameters A-A_0_ for UV-Vis absorption and F-F_0_ for FL intensity. The UV-Vis absorption intensity at characteristic wavelength and FL intensity exhibited progressive attenuation with increasing triazophos concentration. A linear correlation was observed between the logarithm of triazophos concentration and the UV-Vis absorption variation ratio within 0.05 μg/mL–1 μg/mL, yielding the regression equation $\frac{A-A0}{A0}$ = −0.6945 × log[Triazophos] + 0.4507 (R^2^ = 0.9940) with a LOD of 8.98 ng/mL. Similarly, FL measurements demonstrated linearity from 0.01 μg/mL to 0.5 μg/mL, described by $\frac{F-F0}{F0}$ = −2.7533 × log[Triazophos] + 3.3213 (R^2^ = 0.9916), achieving an improved LOD of 1.72 ng/mL (Figures 4f–i).

Parallel experiments with chlorpyrifos revealed analogous concentration-dependent signal suppression patterns in both UV-Vis and FL detection mode. The UV-Vis absorption variation displayed linear correlation ( $\frac{A-A0}{A0}$ = −0.5847 × log[Chlorpyrifos] + 0.1494, R^2^ = 0.9946) from 0.01 μg/mL to 0.5 μg/mL, while FL analysis produced the regression equation $\frac{F-F0}{F0}$ = −1.8745 × log[Chlorpyrifos] + 0.5645 (R^2^ = 0.9926) within the concentration range from 0.01 μg/mL–1 μg/mL, corresponding to LOD values of 5.14 ng/mL and 7.51 ng/mL, respectively (Supplementary Figures S10-S13). This systematic signal suppression mechanism originated from OPs-induced inhibition of AChE activity, which impeded the enzymatic production of TCh essential for triggering UV-Vis and FL signal recovery. These results conclusively demonstrated the AB-Cu NPs sensor’s capability for quantitative OP detection through dual-mode analytical approaches.

### 3.6 Detection of real samples

To evaluate the sensor’s practical applicability in complex herbal matrices, pesticide recovery tests were conducted using Chenpi samples spiked with triazophos. UV-Vis analysis of Chenpi extracts containing final concentration of 0.05 μg/mL, 0.1 μg/mL and 0.5 μg/mL triazophos demonstrated recoveries ranging from 92.00% to 109.92%. FL detection exhibited comparable accuracy with recoveries between 92.36% and 103.18%, validating the sensor’s reliability in herbal medicine. The minor matrix effects observed confirmed the method’s robustness for OP residue detection in phytochemically complex samples. Among the ten double-blind Chenpi samples, pesticide residues were detected in batches No. 9, 10, 95, 96, 97 and 99. Compared with pre-contaminated samples (No. 9, 10, 95, 96 and 99), demonstrating 90% accuracy (9/10 correct identifications) for the sensor-based screening system (Supplementary Figure S14). In the analysis of 15 batches of Chenpi samples, groups S2410050 and S2404720 exhibited FL intensities lower than those of the blank control group, indicating that AChE activity in the system were inhibited, thereby demonstrating the presence of pesticide residues in these samples (Supplementary Figure S15). Meanwhile, the samples were analyzed by HPLC with results summarized in Supplementary Table S1. Sample S2404720 was found to contain pesticide residues exceeding the safety limit. These results collectively highlight the practical utility of AB-Cu NPs in the real-sample detection of pesticide residues in TCMs.

## 4 Conclusion

In summary, this study designed and developed a dual-mode (UV-Vis/FL mode) sensor based on Cu^2+^/Cu^+^ redox cycling for real-time detection of AChE activity, which was further applied in pesticide residue analysis in TCMs. Firstly, AB-Cu NPs exhibited dual “fast-process” advantages (rapid preparation/quick response) through a green synthesis protocol. Moreover, AB-Cu NPs not only aligned with sustainable development principles but also demonstrated high-throughput screening potential. Interestingly, the sensor enabled dual-mode quantitative analysis of AChE activity and pesticide residues in TCMs, while achieving qualitative assessment of enzymatic activity and pesticide presence through visually observable color changes. Importantly, the practical utility of this method was demonstrated by detecting pesticide residues in complex matrices (e.g., triazophos content in Chenpi). Accordingly, the innovative methodological for pesticide residue monitoring in TCM and the technical strategy emphasizing both “green” and “visualization” aspects has paved a new path for developing next-generation detection methods for pesticide residues in TCM.

## Data Availability

The original contributions presented in the study are included in the article/Supplementary Material, further inquiries can be directed to the corresponding authors.
