# Supplementary material for: Cu2+/Cu+ redox-cycling-driven dual-mode sensor for simultaneous monitoring of acetylcholinesterase activity and pesticide exposure
Source: Front Pharmacol. 2025 Aug 22;16:1640821. doi: 10.3389/fphar.2025.1640821 (PMC12411482; doi:10.3389/fphar.2025.1640821)
Supplement: Supplementary file 1 [file DataSheet1.docx]

Supplementary Material

**Cu^2+^/Cu^+^ Redox-Cycling-Driven Dual-Mode Sensor for Simultaneous Monitoring of Acetylcholinesterase Activity and Pesticide Exposure**

**Sitong Lai^1,2,#^, Kunhui Sun^1,2,#^, Yun Wu^3^, Xueyuan Wu^4^, Yiqi Yan^1,5^, Guojing Liu^1^, Xiaoyi Liu^1^, Yuanyuan Ge^2^, Lina Zeng^2^, Ziyu Guo^2^, Shuhong Wang^2^, Ping Wang^2^, Bing Wang^2,*^, Han Zhang^1,*^, Xie-an Yu^2,*^**

^1^ Institute of Traditional Chinese Medicine, Tianjin University of Traditional Chinese Medicine, Tianjin, China

^2^ Shenzhen Institute for drug Control, Shenzhen, China

^3^ Shenzhen tsumura medicine Co. LTD, Shenzhen, China

^4^ Jiangmen Institute for Drug Control, Jiangmen, China

^5^ Haihe Laboratory of Modern Chinese Medicine, Tianjin, China

^#^ These authors contributed equally.

**^*^ Corresponding authors**: Bing Wang, Email: [wangbingszyj@163.com](mailto:wangbingszyj@163.com); Han Zhang, Email: zhanghan0023@126.com; Xie-an Yu, Email: yuxieanalj@126.com.

# Supplementary Data

# Supplementary Figures


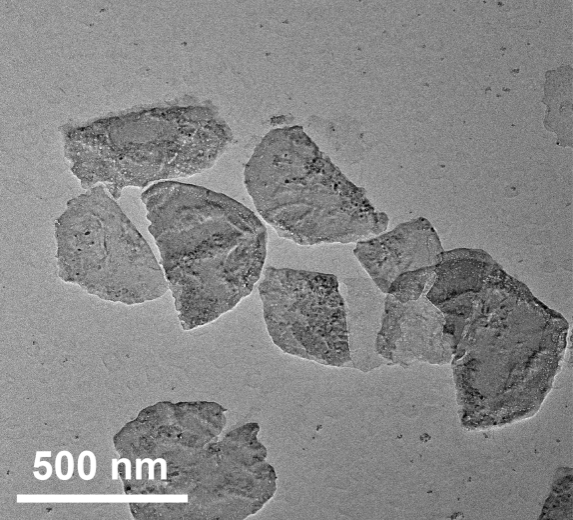


**Figure S1.** TEM image of AB-Cu NPs (Scale bar: 500 nm).





**Figure S2.** Hydrodynamic diameter of AB-Cu NPs over 7 days.





**Figure S3.** FL intensity of AB-Cu NPs over 7 days.





**Figure S4.** FL switching changes (“ON”/“OFF” states) of AB-Cu NPs over 7 days.





**Figure S5.** Relative UV-Vis absorption changes versus ATChCl concentration.





**Figure S6** Relative UV-Vis absorption changes versus ATChCl concentration.





**Figure S7.** Concentration-dependent FL intensity variations.





**Figure S8.** Concentration-dependent FL intensity variations.





**Figure S9.** Comparison of different pesticides.





**Figure S10.** UV-Vis absorption responses to chlorpyrifos pesticide concentrations.





**Figure S11.** UV-Vis-derived calibration curve for chlorpyrifos.





**Figure S12.** Concentration-dependent FL variations of chlorpyrifos.





**Figure S13.** FL calibration curve for chlorpyrifos pesticide.





**Figure S14.** Double-blind evaluation of Chenpi samples.

.





**Figure S15.** Detection of triazophos residues in Chenpi samples.

# Supplementary Tables

**Table S1.** Results of the Chenpi samples by the HPLC

| **Sample Number** | **Pesticide Content (mg/kg)** |
| --- | --- |
| S2410050 | \ |
| S2409650 | \ |
| S2407290 | \ |
| S2406550 | \ |
| S2406090 | \ |
| S2405170 | \ |
| S2404720 | 0.019 |
| S2404830 | \ |
| S2404160 | \ |
| S2403480 | \ |
| S2403340 | \ |
| S2403100 | \ |
| S2402270 | \ |
| S2402010 | \ |
| S2402020 | \ |
